# Clinical Practice of Single‐Molecule Real‐Time Sequencing in the Diagnosis of Spinal Muscular Atrophy

**DOI:** 10.1002/mgg3.70314

**Published:** 2026-10-02

**Authors:** Hua Wei, Ya‐Wen Zheng, Ning‐Ke Zhang, Tie‐Li Gao, Jie‐Xiang Zheng, Tian‐You Li, Shu‐Rong Hong

**Affiliations:** ^1^ Department of Molecular Genetic Center Zhangzhou Affiliated Hospital of Fujian Medical University Zhangzhou China

**Keywords:** single‐molecule real‐time sequencing, *SMN* gene, spinal muscular atrophy, third‐generation sequencing

## Abstract

**Objectives:**

To investigate the distribution proportions of the *SMN1* gene in spinal muscular atrophy (SMA) using single‐molecule real‐time sequencing (SMRT), thereby providing supplementary evidence for SMA genotyping.

**Methods:**

A retrospective analysis was conducted on 771 subjects who underwent both real‐time quantitative PCR and SMRT sequencing tests, followed by statistical analysis.

**Results:**

Among the 771 enrolled individuals, the male‐to‐female ratio was 2:3, with a median age of 31 years. The detection results obtained from real‐time quantitative PCR and SMRT sequencing were consistent. Using SMRT sequencing, the proportions of samples with *SMN1* copy numbers 1, 2, 3, and 4 were 2.33% (18/771), 91.18% (703/771), 6.36% (49/771), and 0.13% (1/771), respectively. For *SMN2*, the proportions with 0, 1, 2, 3, and 4 copies were 6.10% (47/771), 33.07% (255/771), 57.59% (444/771), 2.98% (23/771), and 0.26% (2/771), respectively. The carrier frequency of *SMN1* deletions was 2.33% (18/771). Additionally, we analyzed four SMA patients using SMRT sequencing. The results demonstrate that this technology accurately detects *SMN1* and *SMN2* copy numbers, providing essential information for patient diagnosis and clinical management.

**Conclusion:**

With results fully concordant with qPCR, SMRT sequencing enables accurate detection of *SMN1* and *SMN2* copy numbers, providing robust technical support for population‐based prevention, genetic counseling, and clinical management of SMA.

## Graphical abstract

SMRT sequencing accurately resolves *SMN1* and *SMN2* copy numbers, revealing an SMA carrier frequency of 2.33% and gene distribution patterns. Integrated with case analysis, it provides critical insights for clinical diagnosis and genetic counseling.

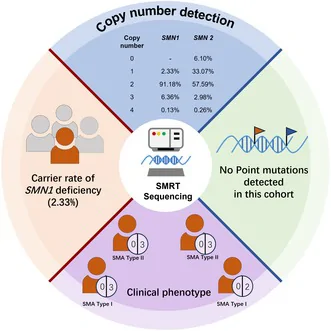

## 1 Introduction

Spinal muscular atrophy (SMA) is an autosomal recessive hereditary progressive motor neuron disease, characterized by muscle weakness, low muscle tone, and progressive atrophy. It is the second most common fatal autosomal recessive genetic disease in infants and young children (Keinath et al. 2021). The pathogenic gene of SMA is Survival Motor Neuron 1 (*SMN1*) (Keinath et al. 2021). The SMN protein encoded by these genes is vital for the survival and functional maintenance of motor neurons. *SMN1* and *SMN2* share a high degree of homology, but a single nucleotide difference at c.840 in Exon 7 of *SMN2* alters pre‐mRNA splicing, resulting in predominantly truncated and nonfunctional SMN protein (Li et al. 2022). Homozygous deletion or mutation of *SMN1* can cause SMA. The carrier frequency of *SMN1* deletions in the general population is approximately 1/50 (Cusin et al. 2003; Li et al. 2020, 2017; Zhang et al. 2024). Given that couples who are both carriers have a 1/4 risk of having an affected child, carrier screening for *SMN1* is of paramount public health significance for preventing the birth of affected children and reducing genetic risk.

Common methods for genetic testing of spinal muscular atrophy (SMA) include quantitative PCR (qPCR) and multiplex ligation‐dependent probe amplification (MLPA). qPCR detects homozygous *SMN1* deletions but not precise copy numbers or point mutations. MLPA detects *SMN1* and *SMN2* copy numbers and exon rearrangements reliably, but cannot determine haplotypes or point mutations (Li et al. 2022). Single‐molecule real‐time (SMRT) sequencing, a third‐generation technology, uses long reads to directly sequence the highly homologous *SMN1/SMN2* regions, enabling haplotype‐level analysis (Bai et al. 2023). It accurately determines copy numbers and key point mutations (e.g., *SMN1* 1 + 1^D^, 2 + 1^D^). For 2 + 0 carriers, SMRT sequencing can distinguish haplotypes in more detail than qPCR or MLPA, thereby aiding the interpretation of pedigree information; however, definitive assignment still relies on family‐based phasing and indirect inference. To assess its clinical utility in carrier screening, we applied it to enrolled samples, using qPCR as references, providing evidence for broader adoption in SMA diagnosis.

## 2 Materials and Methods

### 2.1 Population and Ethics Statement

A total of 771 individuals who underwent routine preconception SMA carrier screening or general health check‐ups at our center from June 2024 to November 2025 were enrolled for carrier frequency estimation (median age 31; male‐to‐female ratio ≈2:3). Additionally, four qPCR‐confirmed SMA patients were included as independent validation cases for diagnostic concordance analysis but were excluded from carrier frequency calculations. This study was approved by the Ethics Committee of Zhangzhou Municipal Hospital (Approval No. 2026LWB061), and written informed consent was obtained from all participants or their legal guardians.

### 2.2 SMRT Sequencing for SMA Genes

Genomic DNA was extracted from peripheral blood. Long‐range PCR was performed to amplify the full‐length *SMN1* and *SMN2* genes and downstream regions using a third‐generation sequencing SMA kit (Berry Genomics, Beijing, China). Amplicons were end‐repaired, phosphorylated, and ligated with barcoded PacBio adapters using T4 DNA ligase to form dumbbell libraries. SMRTbell libraries were prepared with the Sequel II Binding Kit v.3.2 (Pacific Biosciences, CA, USA) and sequenced on a Sequel II CNDx platform (Pacific Biosciences) in CCS mode for 30 h. Subreads were processed using bioinformatics software to generate high‐quality circular consensus sequencing (CCS) reads, which were aligned to the reference genome to identify variants. This method enables simultaneous determination of *SMN1* and *SMN2* copy numbers and full‐length (~28.5 kb) sequencing, with the two genes clearly distinguished by the c.840 site.

### 2.3 Real‐Time Quantitative PCR Method (qPCR)

Extract peripheral blood DNA from the subjects, and follow the instructions provided in the Exon Deletion Detection Kit for *SMN1* (Tianlong, Xi'an, China) to configure the system for Exons 7 and 8 of *SMN1*. Then, use a real‐time quantitative PCR instrument (Bio‐Rad, CA, USA) to perform amplification. Copy numbers were calculated using the ∆∆Ct method, normalized against a genomic reference and intra‐chip batch controls.

## 3 Results

### 3.1 Overall Statistics of the Selected Samples

A total of 771 individuals were analyzed using both qPCR and SMRT sequencing. The two methods showed fully concordant results across all samples (100% concordance). The SMRT sequencing data were then stratified by *SMN1* and *SMN2* copy numbers, as summarized in Table 1. The most common combination was *SMN1*:*SMN2* copy number ratio of 2:2, accounting for 55.25% (426/771), followed by 2:1 at 29.44% (227/771). The proportions of samples with *SMN1* copy numbers 1, 2, 3, and 4 were 2.33% (18/771), 91.18% (703/771), 6.36% (49/771), and 0.13% (1/771), respectively. A total of 18 individuals were identified as carriers of *SMN1* deletions, yielding a carrier frequency of 2.33% (18/771). For *SMN2*, the proportions of copy numbers 0, 1, 2, 3, and 4 were 6.10% (47/771), 33.07% (255/771), 57.59% (444/771), 2.98% (23/771), and 0.26% (2/771), respectively, with 47 individuals (6.10%) exhibiting homozygous *SMN2* deletions.

**TABLE 1:** Distribution of *SMN1* and *SMN2* copy numbers in the study population.

*SMN1*	*SMN2*	Sample (*n*)	Ratio
1	1	1	0.13%
1	2	5	0.65%
1	3	12	1.56%
2	0	38	4.93%
2	1	227	29.44%
2	2	426	55.25%
2	3	10	1.30%
2	4	2	0.26%
3	0	9	1.17%
3	1	26	3.37%
3	2	13	1.69%
3	3	1	0.13%
4	1	1	0.13%

### 3.2 Case Reports

A total of four qPCR‐confirmed SMA patients were included as independent validation cases for concordance analysis between SMRT sequencing and conventional methods. Among them, Patient 1 was a 16‐year‐old female with severe scoliosis on imaging (Figure 1A). qPCR analysis showed homozygous deletions of Exons 7 and 8 of *SMN1* (Figure 1B). MLPA confirmed zero copies of *SMN1* and three copies of *SMN2* (Figure 1C). SMRT sequencing further validated the *SMN1* copy number loss (0 copies; Figure 1D) and *SMN2* copy number gain (3 copies; Figure 1E). No pathogenic point mutations were detected within the screened regions of the *SMN* genes (Figure 1F,G). The results indicated that patient 1 had a homozygous deletion of the *SMN1*, confirming the diagnosis of SMA. The patient began experiencing symptoms such as tremors in both hands at 12 months of age, but was able to stand with support. The patient presented with flaccid paralysis and scoliosis at the age of 16, having received no standardized treatment during the preceding 15 years. According to literature reports (Mercuri et al. 2012; Verhaart et al. 2017), the patient is diagnosed with spinal muscular atrophy type II.

**FIGURE 1:**
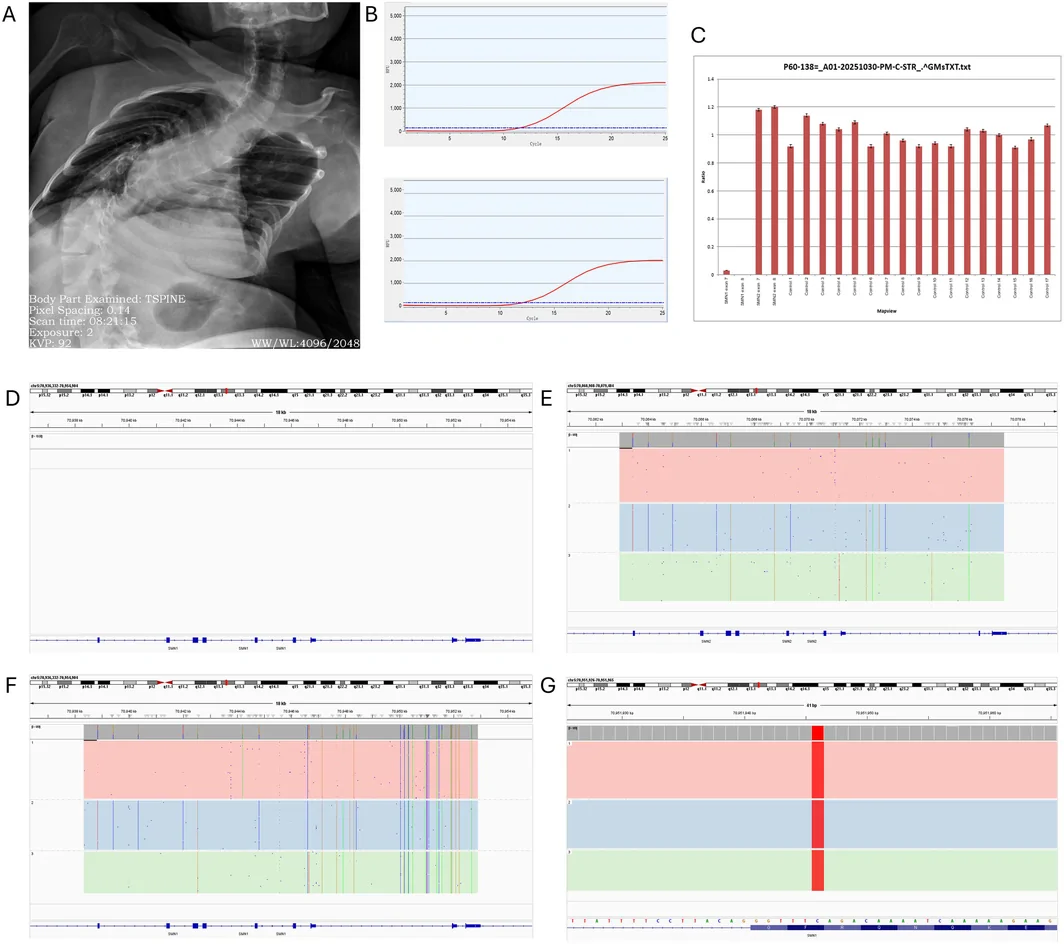
Clinical test results of Patient 1. (A) Chest radiograph of Patient 1 in the frontal position; (B) qPCR results indicating homozygous deletions in Exons 7 and 8 of the *SMN1*; (C) MLPA results indicating zero copies of the *SMN1* and three copies of the *SMN2*; (D) SMRT indicating zero copies of the *SMN1*; (E) SMRT indicating three copies of the *SMN2*. (F) Point mutation detected in the *SMN1* by SMRT sequencing; (G) Point mutation site detected in the *SMN1* by SMRT sequencing.

Patient 2, a 12‐year‐old male, was unable to walk independently at 17 months of age. He presented with scoliosis (Figure 2A). Physical examination revealed proximal upper limb muscle strength graded 2/5, distal strength 3+/5 with reduced muscle tone, and lower limb strength graded 2/5 bilaterally with hypotonia. Knee and ankle jerks were absent. Right hip and knee valgus deformity was noted, with limited adduction of the right lower limb. qPCR analysis demonstrated homozygous deletions of *SMN1* Exons 7 and 8 (Figure 2B). SMRT sequencing further confirmed 0 copies of *SMN1* and three copies of *SMN2* (Figure 2C–F). Based on age at onset and clinical presentation, the patient was diagnosed with spinal muscular atrophy type II.

**FIGURE 2:**
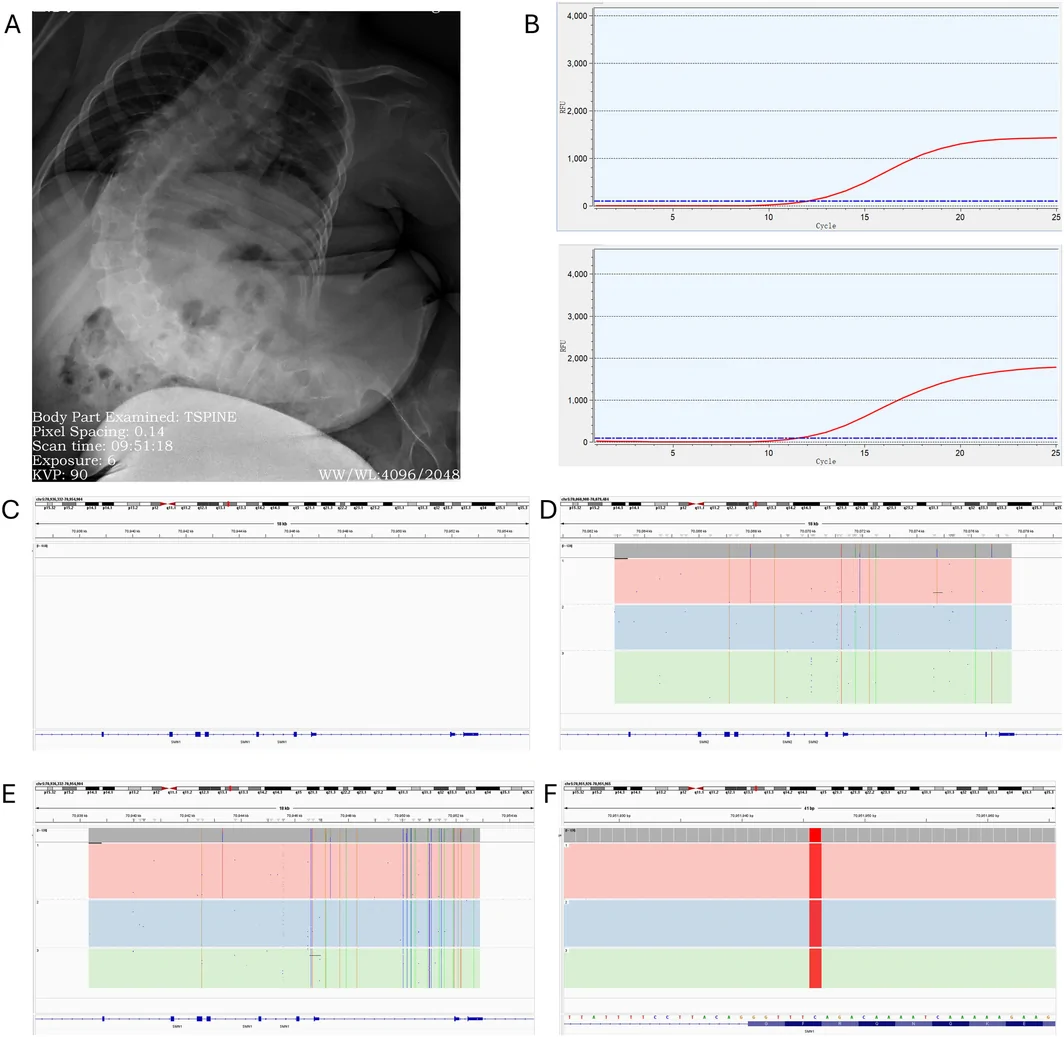
Clinical and genetic findings for Patient 2. (A) Chest radiograph showing scoliosis. (B) qPCR analysis indicating homozygous deletions of *SMN1* Exons 7 and 8. (C) SMRT sequencing confirming zero copies of *SMN1*. (D) SMRT sequencing revealing two copies of *SMN2*. (E) Point mutation analysis by SMRT sequencing. (F) Specific point mutation sites detected by SMRT sequencing.

Patient 3, a 17‐year‐old male, presented with abnormal spinal curvature and absence of normal spinal contours (Figure 3A). Limb abnormalities included flexion contractures of both upper limbs, generalized hypotonia, and paralysis, with upper limb muscle strength graded II and lower limb strength 0. qPCR revealed homozygous deletions of *SMN1* Exons 7 and 8 (Figure 3B). SMRT sequencing further confirmed zero copies of *SMN1* and three copies of *SMN2*, with no pathogenic point mutations detected within the screened regions (Figure 3C–F). Based on comprehensive assessment, the patient was diagnosed with spinal muscular atrophy type I.

**FIGURE 3:**
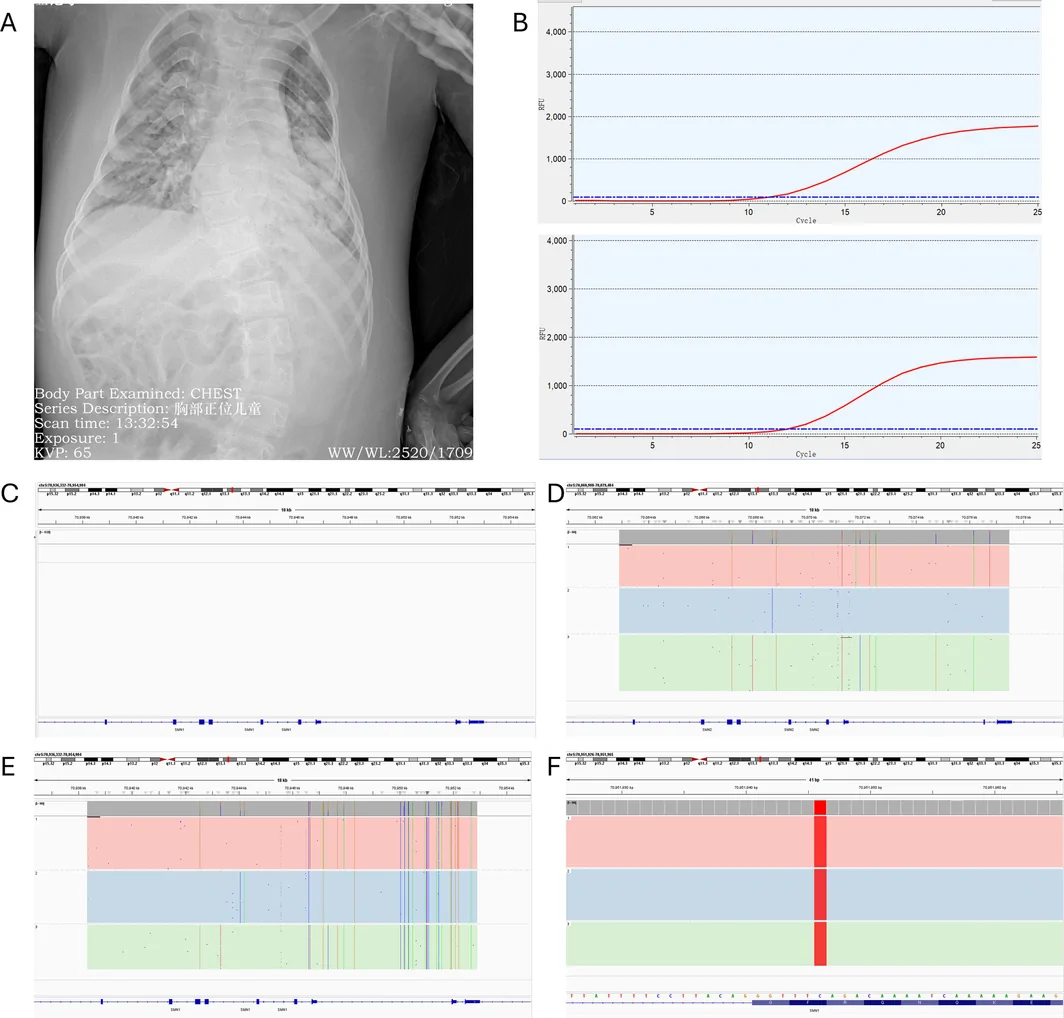
Clinical and genetic findings for Patient 3. (A) Chest radiograph showing absence of normal spinal curvature with abnormal bending. (B) qPCR analysis indicating homozygous deletions of *SMN1* Exons 7 and 8. (C) SMRT sequencing confirming zero copies of *SMN1*. (D) SMRT sequencing revealing three copies of *SMN2*. (E) Point mutation analysis by SMRT sequencing. (F) Specific point mutation sites detected by SMRT sequencing.

Patient 4, a 3‐month‐old male, presented with generalized hypotonia since birth. qPCR analysis confirmed homozygous deletions of *SMN1* Exons 7 and 8 (Figure 4A). SMRT sequencing further validated zero copies of *SMN1* and two copies of *SMN2*, with no pathogenic point mutations detected within the screened regions (Figure 4A–D). Electromyography revealed motor and sensory nerve fiber damage involving the bilateral median, ulnar, tibial, and common peroneal nerves. Based on clinical presentation and diagnostic findings, the patient was diagnosed with spinal muscular atrophy type I according to established guidelines (Mercuri et al. 2018).

**FIGURE 4:**
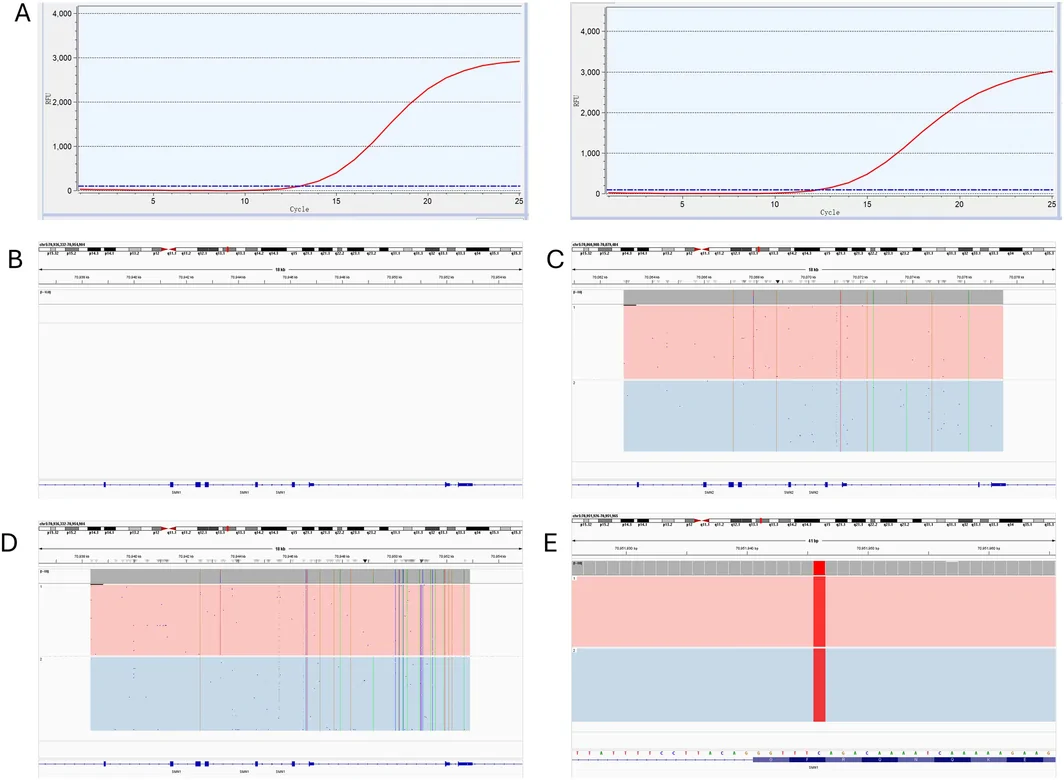
Genetic findings for Patient 4. (A) qPCR analysis indicating homozygous deletions of *SMN1* Exons 7 and 8. (B) SMRT sequencing confirming zero copies of *SMN1*. (C) SMRT sequencing revealing two copies of *SMN2*. (D) Point mutation analysis by SMRT sequencing. (E) Specific point mutation sites detected by SMRT sequencing.

## 4 Discussion

Spinal muscular atrophy (SMA) is a progressive degeneration of spinal anterior horn cells and brainstem motor nuclei caused by the deletion or mutation of *SMN1*. Patients exhibit symptoms such as muscle weakness, hypotonia, and progressive atrophy (Burr and Reddivari 2025). The diagnosis of SMA relies on molecular genetic testing, with commonly used methods including qPCR and MLPA. However, traditional detection methods cannot simultaneously detect *SMN1*/*SMN2* copy number and point mutations in the *SMN1* (Boza‐Moran et al. 2015). The advantages of SMRT sequencing in SMA diagnosis are prominent. SMRT is a single‐molecule sequencing technology without amplification, which has the advantages of ultra‐long read length, no GC bias, and accurate sequencing of complex regions such as highly repetitive sequences. It can comprehensively cover various types of variations, greatly reducing the rate of missed diagnoses (Li et al. 2020). SMRT sequencing has matured and is widely used in genetic testing for diseases such as thalassemia and congenital adrenal hyperplasia (Wirth et al. 2013; Feldkotter et al. 2002). For SMA, which is also a monogenic disease, this technology is equally applicable, and its clinical adoption for this indication remains limited.

This study employed SMRT sequencing to assess the *SMN1* and *SMN2* genotypes in the enrolled population. The results indicated that SMRT sequencing concurred with qPCR detection in determining the presence of copy number deletions. Furthermore, SMRT could provide precise information on the copy number of *SMN1*/*SMN2*. Our study revealed a 2.33% carrier frequency of *SMN1* deletions in the population, which is close to the reported rate of 1.2%–2.2% in the Chinese population (Mercuri et al. 2012). In our study population, two copies of *SMN1* were the most common genotype, observed in 91.18% of individuals. Notably, 6.36% of the population exhibited a *SMN1* copy number of three, and 0.13% carried four copies, indicating the presence of at least two *SMN1* genes on one chromosome in these individuals. This suggests that offspring of such individuals may be silent carriers (2 + 0). However, definitive identification of 2 + 0 status requires pedigree‐based haplotype phasing, which is beyond the scope of the current study. None the less, these findings highlight that individuals with elevated *SMN1* copy numbers may pose underestimated reproductive risks if screened solely by traditional qPCR methods, which cannot infer allele distribution (Long et al. 2024).

SMRT sequencing can simultaneously and directly calculate the copy number of *SMN2* while analyzing *SMN1*. This study found that a high proportion of individuals in the population carry two copies of the *SMN2*, accounting for 57.59%, and one copy accounts for 33.07%. It is worth noting that there are also individuals with three and four copies of the *SMN2*, accounting for 2.98% and 0.26%, respectively. In principle, the *SMN2* can encode SMN protein SMN1, but due to silent mutations in Exon 7 of the *SMN2*, most transcripts are translated into truncated and nonfunctional SMN (SMN‐∆7), which is highly unstable and rapidly degraded (Lorson et al. 1999). Each copy of *SMN2* can only produce approximately 10% of functional protein, with this proportion varying across different tissues (Boza‐Moran et al. 2015; Wirth et al. 2013) Therefore, *SMN2* is considered a modifier gene that acts as a regulator of disease severity. Previous studies have established an inverse relationship between *SMN2* copy number and clinical severity in SMA patients (Feldkotter et al. 2002; Calucho et al. 2018).

In our study, a total of four patients with homozygous deletions of the *SMN1* were detected, among whom three had three copies of the *SMN2*. The SMA types of these three patients were not completely consistent. Two of them developed symptoms 6 months after birth, exhibiting typical clinical symptoms such as being able to sit alone but unable to stand independently and scoliosis. One patient with SMA type I developed symptoms even earlier. This indicates that the clinical phenotypes of patients with three copies of the *SMN2* are variable. Studies have shown that patients with three *SMN2* copies display a broad clinical spectrum, ranging from severe (Type I) to mild (Type IIIc) forms (Dosi and Masson 2024; Butchbach 2016). Calucho et al. (2018) analyzed 1627 SMA patients with three copies of the *SMN2* and found that 15% were Type I SMA, 54% were Type II SMA, and 31% were Type III SMA; 15% presented with Type I, 54% with Type II, and 31% with Type III, further supporting the high variability associated with this copy number. However, studies have reported that within the same clinical subtype, patients with more copies of the *SMN2* exhibit milder symptoms. For example, among Type I patients or infants with *SMN1* deletions, those with three *SMN2* copies exhibit later symptom onset, slower motor decline, and prolonged survival compared to those with two copies. In patients with Type II or Type III SMA, those with only three copies exhibited earlier onset of symptoms, lost the ability to walk earlier, and required ventilator support compared to those with four copies of the *SMN2* (Dosi and Masson 2024). Therefore, given the diverse clinical manifestations of patients with three copies of the *SMN2*, attention should be paid in clinical treatment. In addition, literature reports indicate that among patients with four or five copies of the *SMN2*, 1% and 5%, respectively, exhibit Type I SMA (Dosi and Masson 2024), suggesting that the number of *SMN2* copies is not simply negatively correlated with the severity of the disease. This raises intriguing questions: do different *SMN2* variants possess distinct functional properties, or do modifying mutations within *SMN2* influence its compensatory capacity? The underlying mechanisms remain unclear and warrant further investigation.

SMRT sequencing can accurately identify point mutations, but no such variations were detected in the population included in this study. Literature reports indicate that among SMA carriers, *SMN1* point mutations (1 + 1^D^ or 2 + 1^D^) account for 2% (Qu et al. 2016; Mailman et al. 2001; Hendrickson et al. 2009; Smith et al. 2007). This phenomenon may reflect the true low incidence of these variations in the population, but it also suggests that the current sample size may not be sufficient to make robust statistical inferences about such rare events. Therefore, expanding the scope of detection in subsequent work is crucial for accurately assessing the distribution of such rare genotypes and the screening efficacy of this technology.

## 5 Conclusion

SMRT sequencing not only enables precise detection of *SMN1* and *SMN2* copy numbers but also facilitates simultaneous detection of multiple gene variants. The detection of pathogenic genes in SMA has evolved from single‐site detection to whole‐genome detection. The widespread application of SMRT sequencing is expected to significantly enhance the coverage and accuracy of screening, providing stronger technical support for population prevention and genetic counseling of SMA. Future large‐scale studies will further validate its comprehensive efficacy in detecting rare genotypes of SMA.

## Author Contributions

**Hua Wei:** conceptualization, methodology, writing – original draft, data curation, investigation. **Ya‐Wen Zheng:** conceptualization, methodology, data curation, writing – original draft, investigation. **Ning‐Ke Zhang:** methodology, resources, writing – original draft. **Tie‐Li Gao:** investigation, writing – original draft. **Jie‐Xiang Zheng:** investigation, writing – original draft. **Tian‐You Li:** supervision, writing – review and editing, project administration. **Shu‐Rong Hong:** supervision, resources, writing – review and editing, project administration, funding acquisition.

## Funding

This work was supported by the Natural Science Foundation of Zhangzhou, Fujian, China (grant numbers ZZ2023J27). The funders had no role in the study design, data collection and analysis, decision to publish, or preparation of the manuscript.

## Ethics Statement

This study was approved by the Institutional Review Board (IRB) of Zhangzhou Affiliated Hospital of Fujian Medical University (2026LWB061), and written informed consent was obtained from the patients.

## Conflicts of Interest

The authors declare no conflicts of interest.

## Data Availability

The data that support the findings of this study are available on request from the corresponding author. The data are not publicly available due to privacy or ethical restrictions.
